# The role of cysteines in the structure and function of OGG1

**DOI:** 10.1074/jbc.RA120.016126

**Published:** 2020-11-22

**Authors:** Katarina Wang, Marah Maayah, Joann B. Sweasy, Khadijeh S. Alnajjar

**Affiliations:** 1 Department of Therapeutic Radiology, Yale School of Medicine, New Haven, Connecticut, USA; 2 Department of Cellular and Molecular Medicine, The University of Arizona Cancer Center, Tucson, Arizona, USA

**Keywords:** 8-oxoguanine glycosylase, base excision repair, DNA repair, cysteine oxidation, oxidative stress, 8-oxoG, 8-oxoguanine, BER, base excision repair, IAEDANS, 5-({2-[(iodoacetyl)amino]ethyl}amino)naphthalene-1-sulfonic acid, NEM, N-ethylmaleimide, OGGI, 8-Oxoguanine glycosylase, PUA, α, β-polyunsaturated aldehyde, THF, tetrahydrofuran

## Abstract

8-Oxoguanine glycosylase (OGG1) is a base excision repair enzyme responsible for the recognition and removal of 8-oxoguanine, a commonly occurring oxidized DNA modification. OGG1 prevents the accumulation of mutations and regulates the transcription of various oxidative stress–response genes. In addition to targeting DNA, oxidative stress can affect proteins like OGG1 itself, specifically at cysteine residues. Previous work has shown that the function of OGG1 is sensitive to oxidants, with the cysteine residues of OGG1 being the most likely site of oxidation. Due to the integral role of OGG1 in maintaining cellular homeostasis under oxidative stress, it is important to understand the effect of oxidants on OGG1 and the role of cysteines in its structure and function. In this study, we investigate the role of the cysteine residues in the function of OGG1 by mutating and characterizing each cysteine residue. Our results indicate that the cysteines in OGG1 fall into four functional categories: those that are necessary for (1) glycosylase activity (C146 and C255), (2) lyase activity (C140S, C163, C241, and C253), and (3) structural stability (C253) and (4) those with no known function (C28 and C75). These results suggest that under conditions of oxidative stress, cysteine can be targeted for modifications, thus altering the response of OGG1 and affecting its downstream cellular functions.

More than 100 different oxidized lesions have been identified in DNA (1, 2). The most frequently occurring oxidized DNA lesion is 8-oxoguanine (8-oxoG), with an estimated 2400 lesions at steady-state levels and up to 10,000 under conditions of oxidative stress (3, 4, 5, 6). Because these lesions can form in regulatory regions of the genome, such as promoters and telomeres, they can have an impact on genomic stability and cellular homeostasis (7, 8). The accumulation of 8-oxoG is used as a biomarker for diseases, such as cancer (9, 10), Parkinson’s disease (11), systemic lupus erythematosus (SLE) (12, 13), and rheumatoid arthritis (RA) (14).

8-Oxoguanine glycosylase (OGG1), which has a canonical function in the base excision repair (BER) pathway, can efficiently recognize and excise 8-oxoG (15, 16), binds to the oxidized lesion and catalyzes the cleavage of the N-glycosidic bond to release the oxidized base and create an abasic site (17, 18), and has also been shown to catalyze the cleavage of the DNA backbone by forming a Schiff base intermediate, resulting in the formation of a DNA nick with a 5’ phosphate and 3’ ⍺, β-polyunsaturated aldehyde (PUA) ends. The DNA intermediate is then passed on to APE1, which removes the PUA group so that polymerase beta can incorporate a nucleotide. Finally, the nick is sealed by ligase III/XRCC1 (19).

In addition to its function in DNA repair, OGG1 has been implicated in gene transcription (8, 20, 21), telomere maintenance (7), cell signaling (22, 23), and formation of DNA–RNA hybrids (24). Specifically, OGG1 has been shown to bind 8-oxoG in G-rich promoter regions and regulate the expression of certain oxidative stress–response genes. The binding of OGG1 to promoter regions may have several consequences depending on whether this binding is productive (leads to enzymatic activity) or nonproductive (no enzymatic activity). For example, the nonproductive binding of OGG1 to promoter regions leads to increased expression of certain proinflammatory genes (*CXCL1*, *CXCL2*, *CCL20*, *IL1B*, *TNFA*), indicating that OGG1 glycosylase activity is not necessary for this function (25, 26, 27). On the other hand, productive binding leading to glycosylase activity has been shown to be required for the regulation of hypoxia-induced genes and DNA repair genes (*VEGF*, *NTHL1*, *NEIL3*) (21, 28). After OGG1 binds and forms an abasic site, DNA is rearranged into a G-quadruplex to expose the abasic site. APE1 is recruited but nonproductively binds the abasic site, leading to transcriptional activation of the aforementioned genes. Another possible outcome of productive binding is the formation of a DNA break, which has been shown to be important for the regulation of *BCL2* expression (20).

Because OGG1 plays a central role in maintaining cellular homeostasis under oxidative stress, it is important to have a full understanding of the effect of oxidation on OGG1. In addition to DNA, proteins involved in the cellular response to stress such as OGG1 can also be oxidized, specifically at cysteine residues. Cysteine is a unique amino acid that is highly conserved but can be targeted for modification by oxidation due to the high reactivity of the sulfur atom (29). It has been shown that the oxidation of cysteine increases the functional diversity of proteins by two orders of magnitude (30). Thus, it is important to understand the role of cysteines in the structure and function of OGG1.

Previous studies have suggested that the glycosylase activity of OGG1 is sensitive to specific environmental toxins, like cadmium (31) and arsenic (32). Specifically, the excision efficiency of 8-oxoG by OGG1 is decreased in the presence of cadmium or arsenic, both of which have high affinity for binding to the sulfhydryl group of cysteine (33). This decrease in efficiency indicates that cysteines are important for regulating the various catalytic functions of OGG1 in order to maintain cellular homeostasis under oxidative stress.

OGG1 contains eight cysteine residues that are highly conserved across species (Fig. 1). C28 and C75 are located in the N-terminus, and C253 and C255 are located in the C-terminus. C241, C253, and C255 are within the highly conserved helix–hairpin–helix motif of the enzyme. In this study, we created eight mutants, each containing a single-site cysteine-to-serine mutation. This mutation was chosen to mimic the size of cysteine whilst replacing the sulfhydryl (SH) group with a hydroxyl (OH) group in serine. In each of the eight mutants, we tested DNA binding, glycosylase and lyase activities, and thermal stability. Our results shed light on the importance of certain cysteine residues in the structural and functional characteristics of OGG1. We propose that the modification of certain cysteines can lead to a mild loss of OGG1’s binding affinity to the DNA lesion, in addition to a change in its glycosylase function, and/or its thermal instability, all of which can regulate downstream functions of OGG1. Our results indicate that the cysteines in OGG1 fall into four functional categories: those that are necessary for (1) glycosylase activity (C146 and C255), (2) lyase activity (C140S, C163, C241, and C253), (3) structural stability (C253) and (4) those with no known function (C28 and C75).

**Figure 1:**
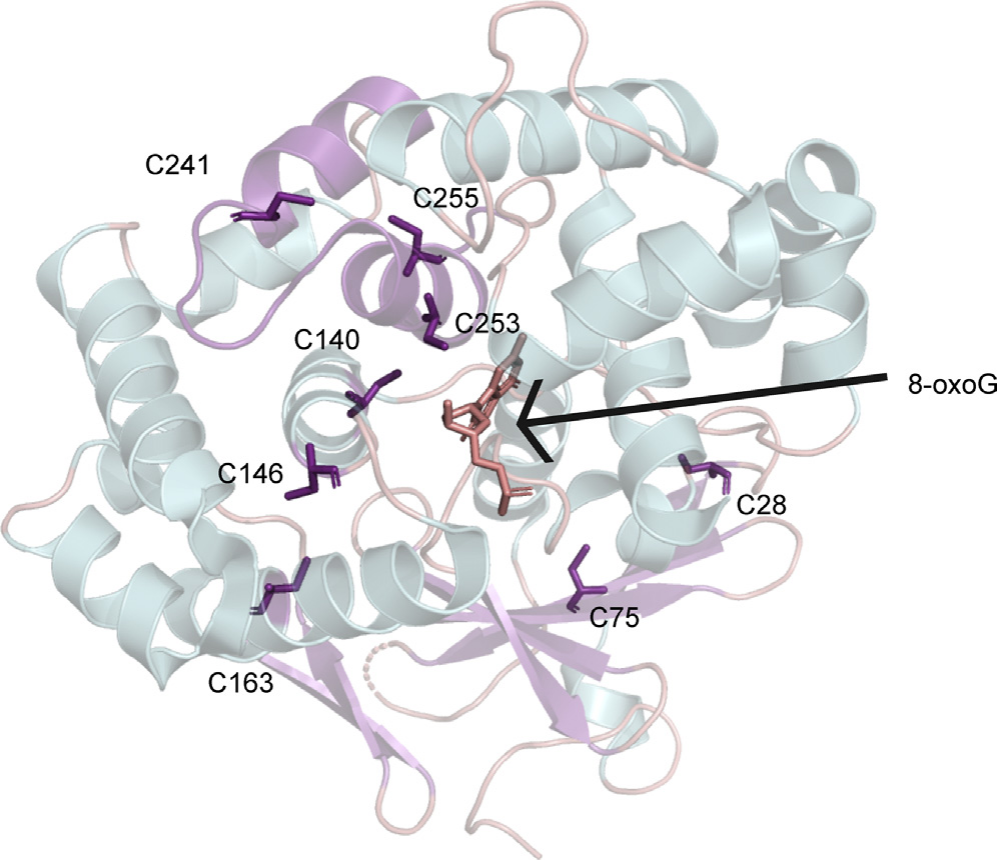
**Cysteines in OGG1 are located throughout the three-dimensional structure.** Crystal structure of OGG1 (PDB: 2NOL) representing the location of the cysteines in *purple*. Also, in *green* is the 8-oxoG lesion flipped out of the DNA duplex (not shown) into the active site of OGG1. PDB, Protein Data Bank.

## Results

### Cysteine modification inhibits glycosylase activity

**Figure 2:**
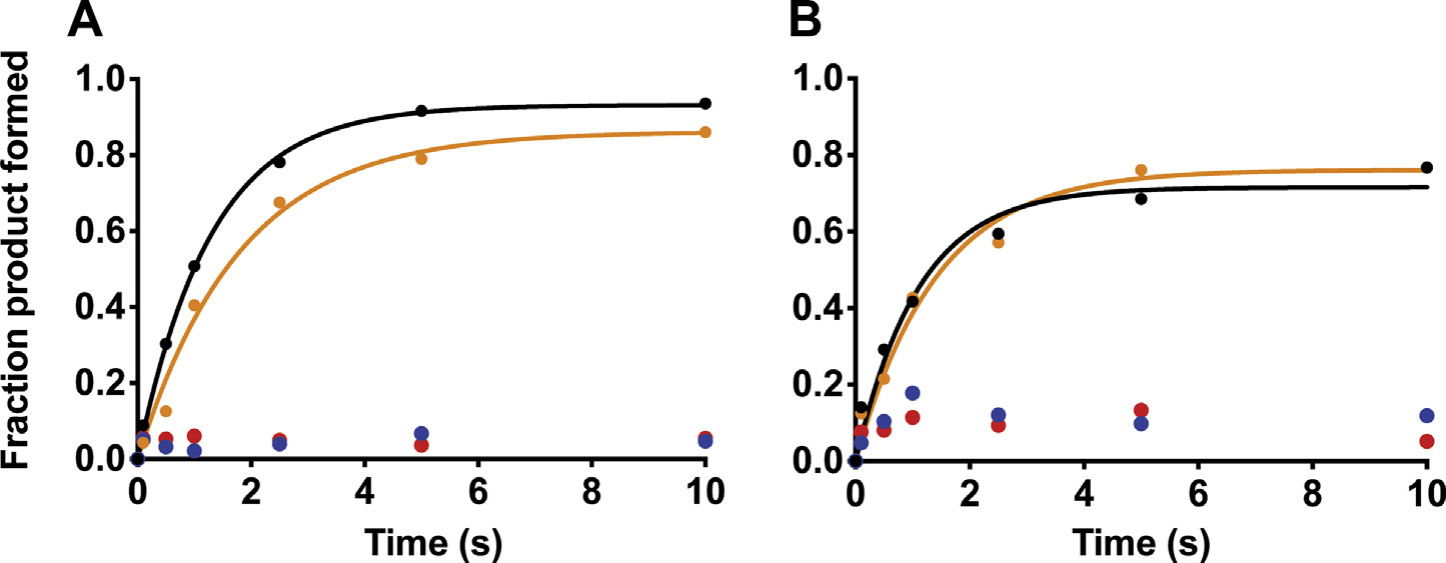
**Cysteine modification decreases glycosylase activity of OGG1.** Modifying the cysteines of OGG1 by varying concentration of *A*, NEM or *B*, IAEDANS results in the loss of glycosylase activity. No treatment control is shown in *black*, 0.1 mM in *orange*, 0.25 mM in *blue*, and 0.5 mM in *red*. IAEDANS, 5-({2-[(iodoacetyl)amino]ethyl}amino)naphthalene-1-sulfonic acid; NEM, N-ethylmaleimide.

OGG1 contains eight cysteine residues. However, little is known about their role in the activity of OGG1. To identify whether OGG1 contains reactive cysteines and whether they are important for activity, we treated OGG1 with N-ethylmaleimide (NEM) and tested the effect of blocking the potentially reactive cysteines on glycosylase activity (Fig. 2*A*). NEM is specifically reactive with the thiol group of cysteine and blocks the sulfhydryl group. In the presence of 0.25 mM NEM (10× molar excess of OGG1), the glycosylase activity of OGG1 is completely abolished. This finding indicates that OGG1 contains reactive cysteine residues that are essential for glycosylase activity. Next, 5-({2-[(iodoacetyl)amino]ethyl}amino)naphthalene-1-sulfonic acid (IAEDANS), a thiol-reactive fluorescent molecule, was combined with OGG1 to quantify the least number of reactive cysteines required to inhibit the activity. The absorbance and extinction coefficient of IAEDANS (ε_336_ = 6100 M^−1^ s^−1^) and OGG1 (ε_280_ = 37,000 M^−1^ s^−1^) were used to identify the labeling ratio. In the presence of 0.25 mM IAEDANS (10× molar excess of OGG1), two cysteine residues were labeled, which was sufficient to inhibit the glycosylase activity (Fig. 2*B*). These results provide evidence that cysteines are important for the activity of OGG1.

### Cysteine mutants of OGG1 bind to the 8-oxoG DNA substrate with variable affinities

**Figure 3:**
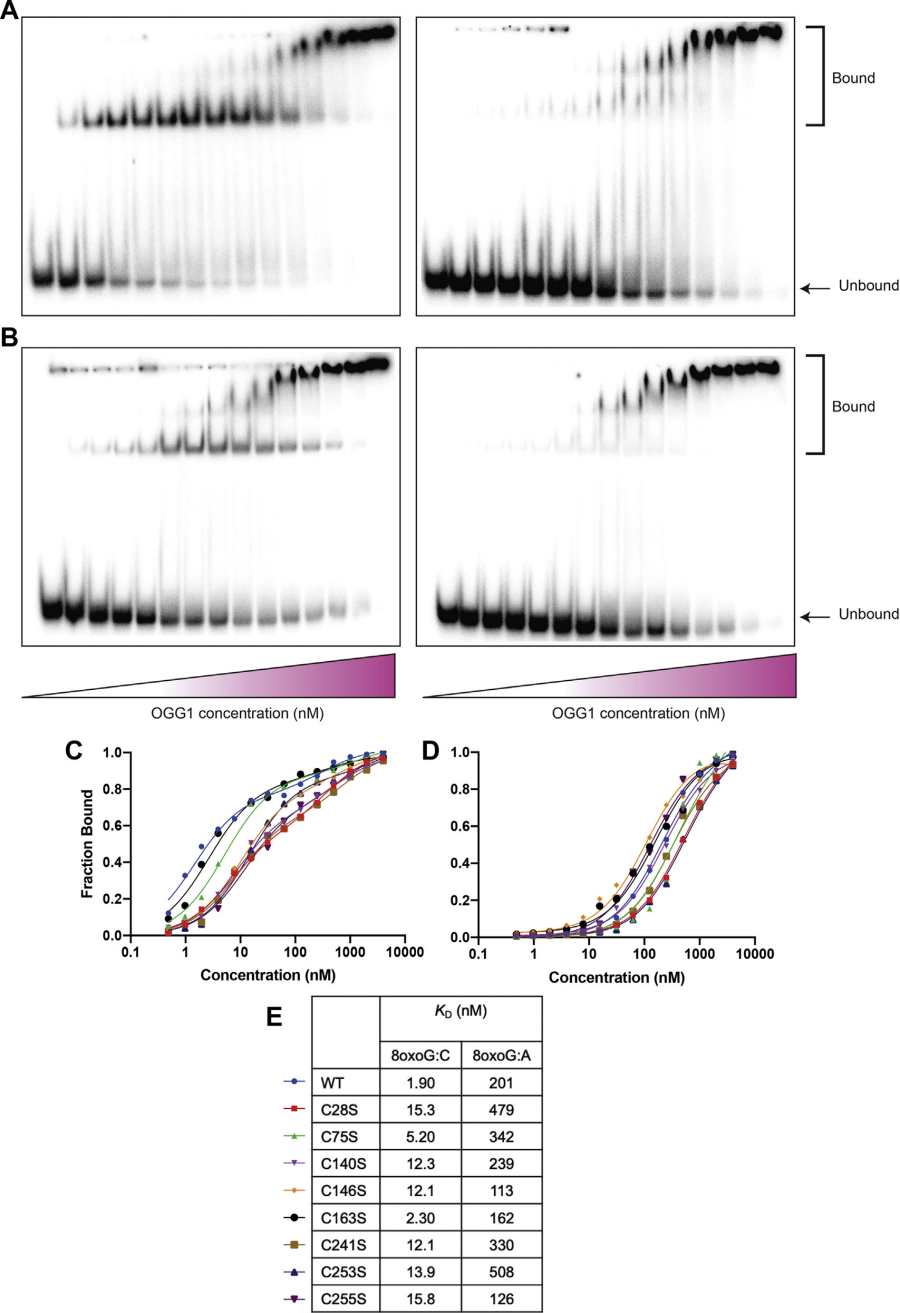
**Various cysteine-to-serine mutants result in an increase in the DNA dissociation constant.** Representative electromobility gel shifts for *A*, WT OGG1 and *B*, C255S binding to their DNA substrate containing 8-oxoG lesion with either templating cytosine (*left gel*) or templating adenosine (*right gel*). Fraction DNA bound was plotted as a function of OGG1 concentration and fitted to a sigmoidal equation for the templating *C*, cytosine and *D*, adenosine in order to extract the apparent binding constants for each of the mutants relative to the WT (*E*).

In order to understand the role of cysteines in DNA binding, an electrophoretic mobility shift assay was performed using a DNA substrate containing the 8-oxoG lesion. The assay was performed at 4 °C in order to prevent any catalytic activity (34). The results indicate that WT OGG1 binds tightly to its DNA substrate containing the lesion opposite a templating cytosine with a dissociation constant of 1.9 nM (Fig. 3, *A* and *C*). However, most of the cysteine mutants bind to the DNA with an altered affinity. Specifically, mutations at positions 28, 140, 146, 241, 253, and 255 have an ∼10-fold increase in the dissociation constant. The decreased binding affinity in C28S was unexpected due to its distal location from the DNA binding site and the active site. This finding indicates that C28 may influence DNA binding through long-range interactions. The increase in the dissociation constant of both C253S and C255S is expected due to their proximal location in the helix-hairpin-helix motif near the DNA binding site. Specifically, C253 has been shown to be part of the active site, which interacts with the lesion once it is extruded into the active site (35). Lastly, not much is known about the role of C140, C146, and C241 in the function of OGG1. Because they are located near the DNA binding pocket, a mutation at any of these positions is expected to have an effect on the dissociation constant despite the lack of direct contact with DNA. Our findings show that mutations at these positions do, in fact, cause an increase in the dissociation constant. Thus, we provide evidence here that although most cysteines lack direct interactions with DNA, they are important for DNA binding, with C28, C140, C146, C241, C253, and C255 being especially crucial for this function.

In addition to exhibiting substrate specificity for the 8-oxoG lesion over other oxidized lesions, WT OGG1 also preferentially binds to 8-oxoG:C (specific binding) over 8-oxoG:A (nonspecific binding) substrates (17). This selectivity is a mechanism to prevent the repair of mispaired 8-oxoG:A and the propagation of error. In this study, we investigate whether the cysteine mutations influence the specific and nonspecific binding of OGG1. As previously mentioned, our results show that compared with WT, OGG1 with cysteine mutations has a lower specific binding affinity (dissociation constants of 1.9 nM for WT and 2.3–15.8 for the mutants). On the other hand, the nonspecific binding affinities (to 8-oxoG:A) of the mutant OGG1 compared with that of the WT do not differ considerably (dissociation constants of 113–507 nM and 201 nM, respectively) (Fig. 3, *B*–*C*). The results indicate that OGG1 binds specifically to its substrate and that the cysteine mutations only influence specific binding but not nonspecific binding.

### C146S and C255S have decreased glycosylase activity

**Figure 4:**
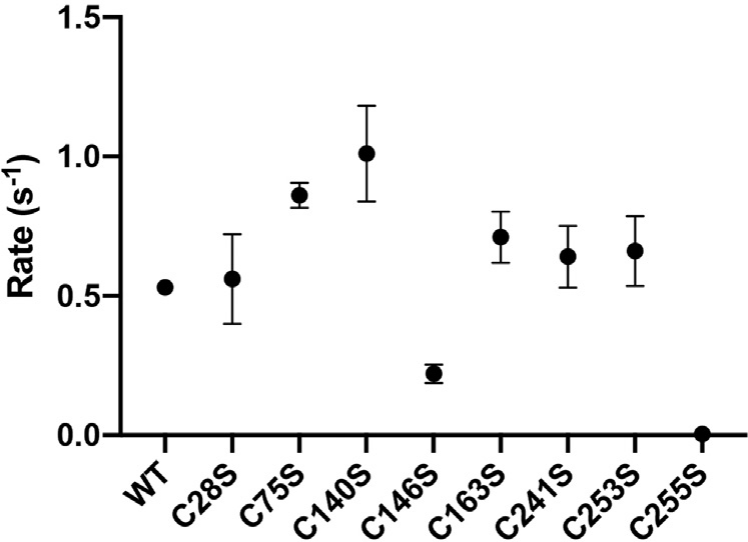
**Certain cysteine-to-serine mutants affect the glycosylase activity of OGG1.** The rate of glycosylase activity for each of the cysteine-to-serine mutants of OGG1 as measured by quench flow. C146S and C255S cause a severe inhibition in the rate of glycosylase activity.

The ability of each of the cysteine mutants to catalyze the removal of 8-oxoG and create an abasic site was tested in the presence of excess OGG1 (10× molar excess relative to DNA) to ensure that the reaction is not limited by percent active enzyme and by the slow rate of DNA dissociation (36). The reaction was performed using the quench-flow apparatus, which rapidly mixes OGG1 with the DNA and quenches the reaction with NaOH at selected time points (the alkaline hydrolysis reaction cleaves the DNA backbone at abasic sites). The lyase activity of OGG1 is the rate-limiting step in the reaction of OGG1; therefore, NaOH is used to ensure that the observed rate is not limited by the rate of the lyase activity (37). Fraction product formed was plotted as a function of time and fitted to a single exponential equation. The results indicate that WT OGG1 catalyzes glycosylase activity with a rate of 0.53 s^−1^ (Fig. 4).

The results indicate that the C146S mutant has a slow glycosylase rate (0.22 s^−1^). Thus, a modification at this site can decrease the glycosylase activity. C146 is located near the active site, but there are no direct interactions with the lesion and DNA. The shortest distance between C146 and the lesion is 5 Å, with no apparent intermediate molecules between them. Therefore, C146 might be important for stabilizing the extruded lesion or it might be involved in recognizing and flipping the lesion out of the duplex.

The most severe loss in activity resulted from the C255S mutation, with a rate of 0.004 s^−1^. This finding corresponds with the location of C255 near the active site, which likely makes C255 essential for the glycosylase activity. Both C253 and C255 are located within the same helix, with the closest distance being 4.3 Å between the pair. As previously discussed, C253 has been shown to be a part of the active site, important for binding the lesion once it is extruded out of the DNA duplex and into the active site (35). Previous work has shown that a mutation of C253 to leucine or isoleucine decreases the activity severely (37). However, our results indicate that a mutation to serine has no effect on the glycosylase activity. We hypothesize that the bulkiness of the group at position 253 in previous studies affected OGG1 glycosylase activity, as leucine and isoleucine have bulkier side chains than cysteine and serine.

Interestingly, the C140S mutant led to an increase in the glycosylase rate (1.0 s^−1^). The mechanism is unclear because of the lack of direct interactions between C140 and the active site. This finding provides evidence that there could be several consequences to the modification of cysteines depending on the site and type of modification (bulkiness and charge).

### Mutation at the C-terminus cysteines decreases lyase activity

**Figure 5:**
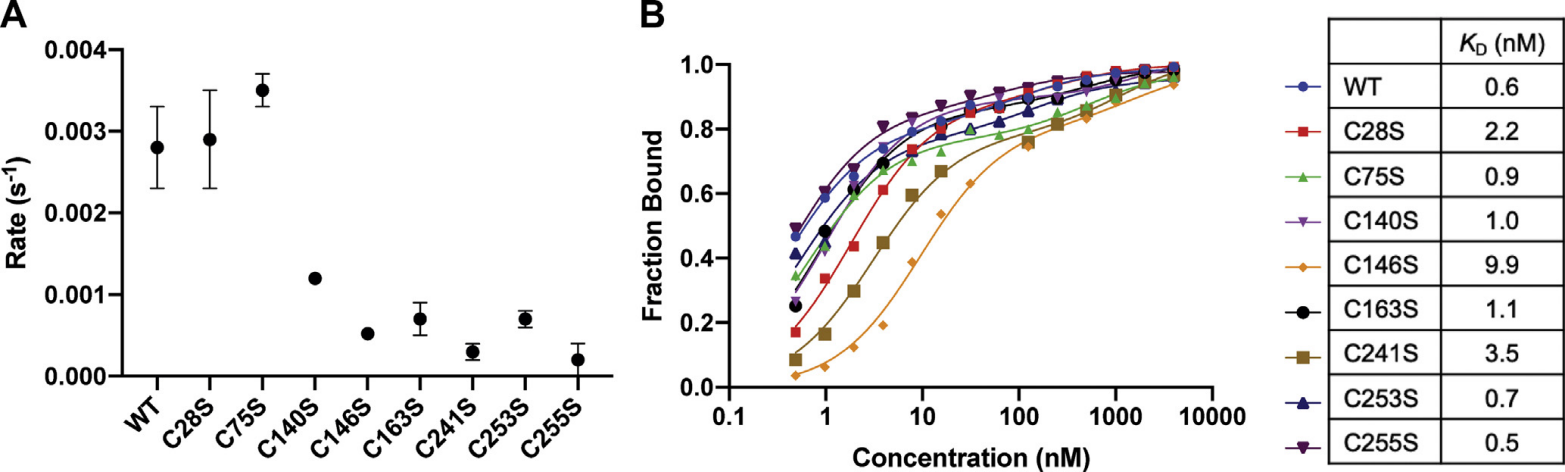
**Various cysteines are important for the lyase activity.***A*, the rate of lyase activity is dependent on many of the C-terminal cysteines. *B*, the effect on lyase activity is independent of the ability of OGG1 to bind to the DNA containing the abasic site (THF). THF, tetrahydrofuran.

The lyase activity of OGG1 results in the formation of a nicked DNA backbone at the 5’ end of the lesion, leaving a 3’ PUA and a 5’ phosphate. In the BER pathway, the PUA group is further processed by APE1 to form a 3’OH, which is the substrate for the subsequent nucleotide incorporation reaction by polymerase beta. The lyase activity was tested by mixing OGG1 with the 8-oxoG–containing DNA substrate at 37 °C and quenching the reaction at each time point with formamide dye followed by incubation at 85 °C for 5 min. The results in Figure 5*A* indicate that WT OGG1 catalyzes the lyase reaction at a rate of 0.0028 s^−1^. Even though the rate of lyase activity is slow, the long residence time of the protein on DNA due to the slow rate of DNA dissociation provides ample time for the enzyme to perform its lyase activity. All cysteine-to-serine mutants clustering near the active site at the C-terminus have a decreased lyase activity compared with WT, including C140S, 146, 163, 241, 253, 255 (Fig. 5*A*). This finding indicates that the lyase activity is highly sensitive to the status of cysteines.

To ensure that the decrease in lyase activity is not resulting from a change in DNA binding to the abasic site, we measured the apparent binding affinity of the enzyme to a DNA substrate containing tetrahydrofuran (THF), which is a stable mimic of the abasic site. Our results indicate that WT OGG1 has a dissociation constant of 0.6 nM. Although a few cysteine mutants have higher dissociation constants, specifically C28S (2.2 nM), C146S (9.9 nM), and C241S (3.5 nM), most mutants have dissociation constants that are very similar to WT and they maintain their high binding capacity to the THF-containing substrate (Fig. 5*B*). This finding indicates that the decrease in lyase activity is not due to a change in DNA binding.

### Certain cysteine residues affect the thermal stability of OGG1

**Figure 6:**
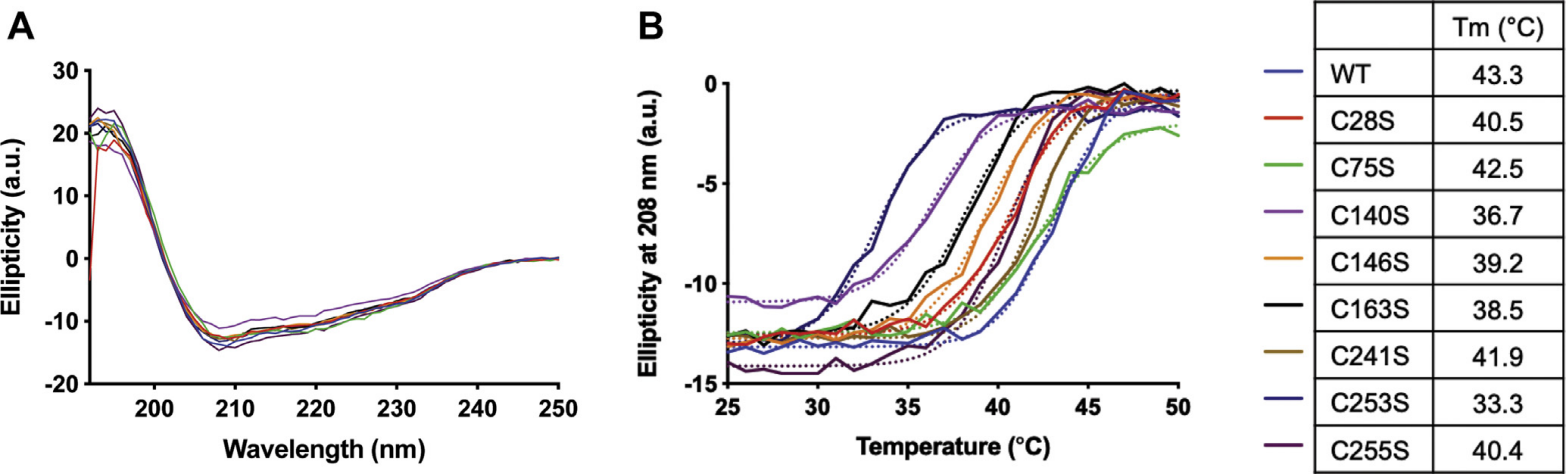
**The thermal stability of OGG1 is dependent on certain cysteines.***A*, CD spectra of OGG1 mutants compared with the WT at 25 °C. Ellipticity of the ⍺-helical structure was measured at 208 nm as a function of temperature. *B*, the melting temperature (*T*_m_) was estimated as the temperature at which 50% of the secondary structure has denatured. The WT has a *T*_m_ of 43.1 °C; each of the mutants had a different effect on the extent of stability.

In order to test the role of each of the cysteine residues in maintaining the structural stability of OGG1, protein ellipticity at 208 nm was measured as a function of temperature using circular dichroism spectrometry (Fig. 6). This wavelength was used because the protein is mostly ⍺-helical (Fig. 6*A*). The WT has a melting point (T_m_) of 43 °C, similar to previous reports (38). The most significant loss in stability resulted from the C140 and C253 mutations, leading to a T_m_ of less than 37 °C (36.3 and 33.6 °C, respectively). C140 is located within an ⍺-helix, and a mutation at this position may lead to instability. C253 is located at the active site and has been shown to be important for the catalytic activity (35). These results indicate that cysteine modifications may influence the structural stability of the enzyme.

## Discussion

OGG1 is a key enzyme involved in various processes and is responsible for maintaining cellular homeostasis in response to oxidative stress. In this study, we propose that the cysteines in OGG1 act to regulate the various functions of the protein, especially under conditions of oxidative stress. In order to study the role of cysteines in the function of the protein, we created individual cysteine-to-serine mutants and biochemically characterized them. The cysteines of OGG1 fall into four functional categories: those that are necessary for (1) glycosylase activity (C146 and C255), (2) lyase activity (C140S, C163, C241, and C253), (3) structural stability (C253) and (4) those with no known function (C28 and C75). Thus, a modification at specific cysteine positions may lead to different consequences in the cell.

### The importance of DNA binding specificity

As a part of the BER pathway, OGG1 searches the DNA for oxidized lesions and binds specifically to the 8-oxoG lesion that is paired with cytosine (18). The recognition of 8-oxoG:C and removal of the lesion by OGG1 serves two functions. First, the removal of the 8-oxoG lesion prevents the preferred misincorporation of adenine opposite of the 8-oxoG by replicative and repair polymerases. Second, the decreased selectivity for repairing 8-oxoG:A lesions compared with 8-oxoG:C lesions prevents the accumulation of guanine-to-thymine transversion mutations. If the 8-oxoG:A lesion is to be repaired by OGG1, then the polymerase will incorporate a thymine opposite adenine (39, 40, 41, 42). Thus, it is important for OGG1 to maintain its binding specificity under conditions of oxidative stress. Our data show that nonspecific binding is not influenced by the cysteine mutations, indicating that OGG1 with mutated cysteines can continue to preferentially bind 8-oxoG:C lesions over 8-oxoG:A lesions.

### The role of N-terminal cysteines in OGG1

Upon binding to the lesion, OGG1 causes a structural rearrangement in the DNA, resulting in the formation of a 70° bend in order to access the lesion. The base lesion is then extruded out of the duplex DNA and into the active site of OGG1, while N149 and N150 fill the void in the duplex DNA (35). G42 is located in the N-terminus of the enzyme and interacts with the N7 proton of the lesion (35, 43). Thus, perturbations extending through the N-terminus may alter the lesion specificity of the enzyme. Both C28 and C75 are located in the N-terminus of the enzyme. Although these two sites are distant from G42, any instability resulting from a modification at these sites could extend to deter the hydrogen bonding between G42 and N7 of the lesion and decrease the binding affinity. Our results indicate that a mutation at position C28, but not C75, leads to an 8-fold decrease in the binding affinity to the 8-oxoG DNA substrate without affecting the activity. This result suggests that a modification at C28 may indirectly affect DNA binding through long-range interactions. Interestingly, when we purified each cysteine-to-serine mutant, C28 and C75 had low yields (∼10% relative to WT). We hypothesize that an intact and unmodified N-terminus is necessary for the stability of the protein. The dynamic characteristic of the N-terminus is exemplified by the absence of the N-terminus in most Protein Data Bank entries of OGG1, as most entries have truncations in the N-terminus in order to form a stable crystal structure. This absence of the N-terminus indicates that it is a dynamic domain in the structure that influences the stability of the protein.

### C253 is a potential oxidation target that is important for activity

Previous quantum mechanics/molecular mechanics simulations provide evidence that the specificity of OGG1 toward 8-oxoG requires the formation of a dipole in the active site. K249 is shown to be protonated, and C253 is deprotonated in order to stabilize the interaction with the extruded 8-oxoG (43). These results indicate that C253 exists in the reactive ionized form at the active site, which can be further supported by the close proximity of C253 to K261. This proximity may alter its pKa and the stability of the ionized form of C253, indicating that C253 can exist in the ionized form and can be targeted for oxidation. C253 is important for OGG1 specificity and activity because it acts to sandwich the extruded base lesion. Thus, a modification at this position may alter its activity.

The decrease in catalytic activity resulting from a mutation of C253 into leucine or isoleucine indicates that the bulkiness of these aliphatic residues distorts the active site but maintains the ability of OGG1 to recognize the lesion (37). As expected, our results suggest that mutating C253 into serine has little to no effect on the function of the enzyme because serine does not have a bulky side group to inhibit the enzyme. However, larger modifications by oxidation at position C253 may lead to active site distortions.

### C255 and C146 are important for the glycosylase activity

C253 and C255 are located in the helix–hairpin–helix motif of the C-terminus of OGG1, which is a conserved motif within many glycosylases (for example, MPG and MutY) (44). Mutating C253 and C255 into serine did not influence DNA binding; however, a mutation at C255 led to a severe decrease in the glycosylase activity. C253, which directly interacts with the extruded lesion, is located on the same helix as C255. Thus, the decreased glycosylase activity resulting from C255S could occur indirectly through an effect on C253 because of its location in the same helix or by an alternative mechanism. One possibility is that C255 interacts with the alpha carboxyl of A251 (3.2 Å away from C255), which is 3.3 Å away from the active site residue K249. Therefore, C255 has a critical role in the function of OGG1 owing to its proximity to the active site.

In addition to C255, we provide evidence of a novel role of C146 for the glycosylase activity. A mutation at C146 led to a decrease in the glycosylase activity. C146 is located near N149 and N150, which are important for filling the void in the duplex DNA upon extrusion of the lesion out of the duplex and into the active site. This proximity may explain why C146 is essential for the glycosylase activity (35).

### Separation-of-function mutants in OGG1 indicate that lyase activity is highly regulated compared with glycosylase activity

In addition to catalyzing the glycosylase activity to excise the damaged base, OGG1 has a lyase function: it cleaves the DNA backbone 5’ to the abasic site by β-elimination to create a 3’ PUA and 5’ phosphate. The 3’ PUA DNA end does not allow the polymerase to incorporate a nucleotide; thus, APE1 binds to cleave the 3’ PUA and creates a 3’OH substrate for the polymerase. The lyase rate is a slow step; however, DNA dissociation is also slow (0.0028 s^−1^) (36), giving the enzyme enough time to perform the lyase activity. Cysteine at positions 140, 146, 163, 241, 253, and 255 all show a marked decrease in lyase activity compared with the WT enzyme. Because mutating C140, C163, C241, and C253 inhibits lyase activity but not glycosylase activity, we show that lyase activity is highly regulated compared with the glycosylase activity. These results suggest that the lyase activity is important for the response of the protein to oxidative stress.

### Additional applications of cysteines in OGG1

Another factor by which the cysteines could influence the ability of OGG1 to participate in a specific oxidative stress pathway is by affecting protein–protein interactions. Specifically, several BER components such as APE1 and XRCC1 have been shown to interact with OGG1 and facilitate the repair of 8-oxoG. For example, the interaction of OGG1 with XRCC1 or APE1 has been shown to increase the turnover rate of OGG1 by a factor of 3× and 5×, respectively (45, 46). Moreover, OGG1 has been shown to interact with several transcription factors, such as NFκB and SP1 (27). Although this is beyond the data presented in this work, the modification of cysteines can act to alter the interaction with certain proteins.

### Three scenarios for transcriptional activation induced by OGG1

**Figure 7:**
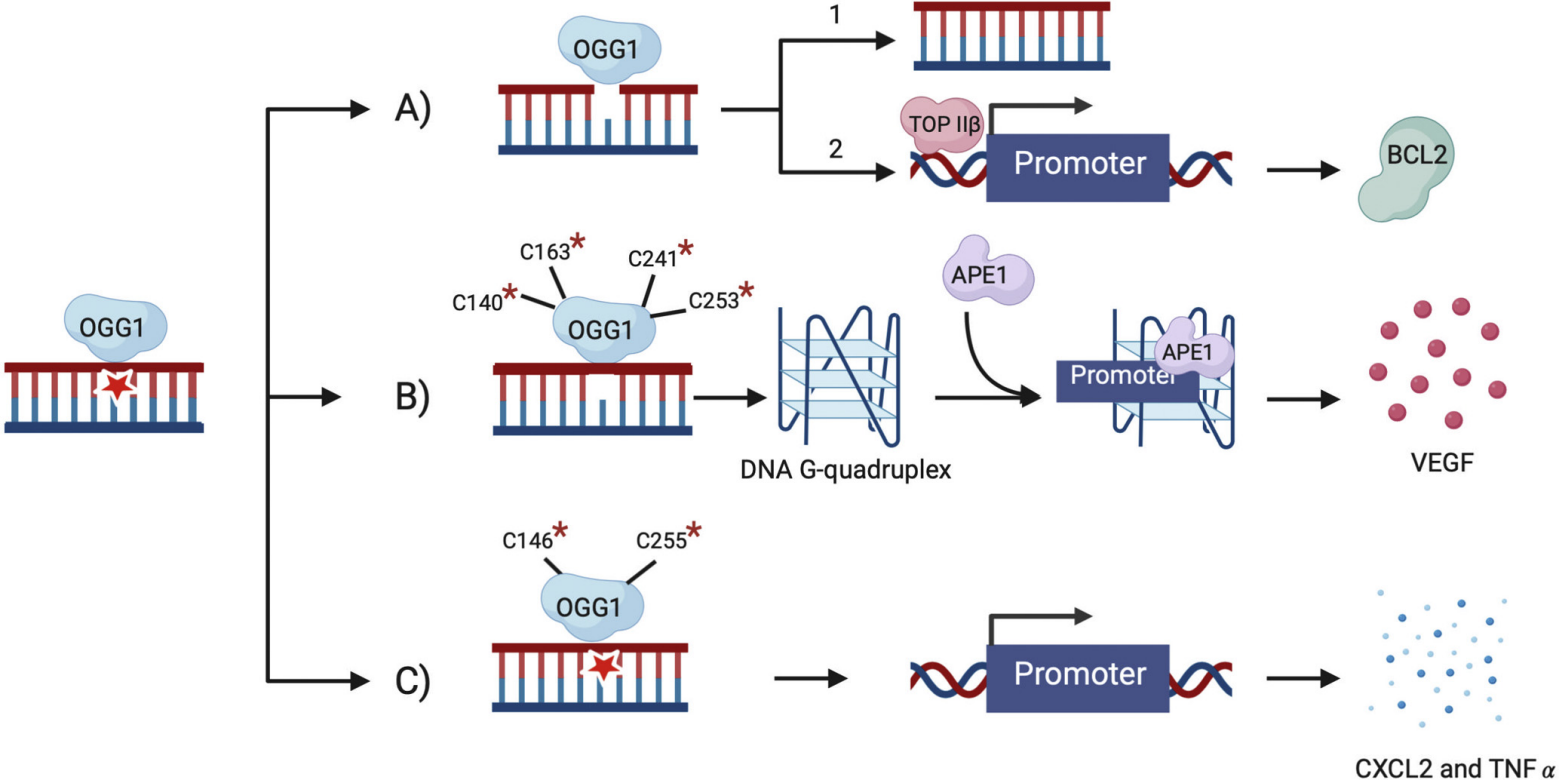
**Three models for transcriptional activation induced by OGG1.***A*, in the absence of cysteine oxidation, OGG1 binds to its substrate and repairs DNA through glycosylase and lyase functions (1) or activates the transcription of *BCL2* (2). *B*, the oxidation of C140, C163, C241, and/or C253 (denoted by asterisks) results in the formation of an abasic site but inhibits the lyase activity. This leads to the formation of a G-quadruplex, which recruits APE1 and activates the transcription of *VEGF*. *C*, the oxidation of C146 and/or C253 inhibits OGG1’s glycosylase and lyase activities, but OGG1 maintains its affinity to bind the oxidized lesion in promoter regions. This induces transcription of proinflammatory genes, such as *CXCL2* and *TNFA*.

In addition to maintaining genomic stability and preventing propagation of mutations through the BER pathway, OGG1 has several other functions in the cell. Most prominently, OGG1 has been shown to bind 8-oxoG in promoter regions of certain genes and alter their expression level (8, 21, 27, 28, 47). However, the requirement for BER activity has been controversial. Specifically, transcriptional activation by OGG1 has been described by three scenarios (Fig. 7).

In the first scenario, the formation of a DNA break triggered by the activity of OGG1 is necessary for the transcriptional activation of *BCL2* because it induces topological changes in the promoter region necessary for its transcription (Fig. 7*A*) (20). The second scenario indicates that OGG1 binds promoter regions, such as hypoxia-response elements, and removes the oxidized legion to create an abasic site. Then, using the abasic site, the DNA structure is reconfigured into a G-quadruplex. This reconfiguration results in the recruitment of APE1 to the abasic site, but with attenuated endonuclease activity (21). This sequence of events leads to the activation of transcription of genes like the vascular endothelial growth factor (Fig. 7*B*). Lastly, the third scenario suggests that only the binding of OGG1 at promoter regions but not its catalytic activity is necessary for the transcriptional activation of various proinflammatory genes, such as *CXCL2* and *TNFA* (Fig. 7*C*) (47). Due to the multifunctional nature of OGG1, a mechanism to regulate its functions is necessary, especially in the context of stress. In this work, we propose the modification of cysteines to be the mechanism by which OGG1 regulates its activities.

Our results, which indicate that cysteines in OGG1 fall into four different categories, can explain how OGG1 cysteine mutants may affect activity in the three scenarios described above. In this work, we observe that cysteines within OGG1 have different effects on its activity. Thus, site-specific modifications can act as a mechanism to regulate the activity of OGG1. For example, in the absence of cysteine modifications, transcriptional activation *via* the first scenario (formation of break) can occur (Fig. 7*A*). Modifications at positions C140, C163, C241, and C253 maintain the glycosylase activity but inhibit lyase activity, potentially leading to the transcriptional activation described in the second scenario, where formation of abasic site is required but nicking of the DNA backbone is attenuated (Fig. 7*B*). Lastly, our results indicate that positions C146 and C255 are important for the glycosylase activity; thus, a modification at C146 or C255 can activate the pathway in the third scenario, where the nonproductive binding of OGG1 is required for transcription activation (Fig. 7*C*). Recent work by Hao *et al.* (48) supports the notion that the activity of OGG1 needs to be regulated in order to steer the function of OGG1 toward either DNA repair or gene transcription.

### Cysteine oxidation as a regulation mechanism in DNA repair

Previous work has shown that OGG1 is targeted for modification in the presence of cadmium and arsenic, both of which lead to increased levels of reactive oxygen species and inhibit the activity of OGG1 (31, 32). Thus, OGG1 can sense oxidative stress through its cysteine residues, leading to the regulation of its activity in response to oxidative stress.

Additional DNA repair proteins from different pathways have been shown to be targeted for oxidative modifications (49). For example, the oxidation of a cysteine residue in the DNA damage sensor, ataxia-telangiectasia mutated (ATM), results in the formation of a cysteine-mediated cross-linked dimer that leads to its activation in the absence of DNA breaks, acting as a sensor for oxidative stress (50). Moreover, the formation of a disulfide bond between cysteines within the N-terminus of the BER scaffolding protein, X-ray repair cross-complementing protein 1 (XRCC1), under oxidative stress conditions has been shown to decrease its binding affinity to polymerase beta (51). Lastly, DNA polymerase gamma has been recently shown to be targeted for oxidative modification, including the oxidation of multiple cysteine residues, leading to inhibition of its exonuclease activity and subsequent decrease in the polymerase replication fidelity (52). These examples provide strong evidence that cysteines can act as a regulatory mechanism in DNA repair and should be further investigated.

### OGG1 cysteine mutations in cancer

Data from the Cancer Genome Atlas on OGG1 indicate that a number of single nucleotide polymorphisms found in patients with cancer result in mutations into cysteine (for example, R97C and R330C), which may further sensitize the protein to oxidation. Specifically, S326C is a frequently occurring mutation in OGG1 that has been associated with various cancers such as lung cancer (53), prostate cancer (54), and breast cancer (55). Functional analysis of S326C indicates that under conditions of oxidative stress, the mutant forms a disulfide bond–induced dimer, which inhibits OGG1 (56). Therefore, studying the effect of oxidation on the function of OGG1 is important for understanding molecular mechanisms of cancer and defining the correlation between oxidative stress and cancer. Lastly, OGG1 inhibition could provide therapeutic benefits by inhibiting the proliferation of cancers with high oxidative stress and sensitizing tumor cells to apoptosis with chemotherapy and radiotherapy (57, 58). Thus, OGG1 is a promising target for cancer diagnosis and treatment.

## Experimental procedures

### Expression and purification of OGG1

OGG1 in pET30 vector with an N-terminal 6x-histidine tag was provided by Dr Sylvie Doublie. Site-directed mutagenesis was used to create the following eight mutants: C28S, C75S, C140S, C146S, C163S, C241S, C253S, and C255S. The protein was expressed in Rosetta (DE3). The Rosetta (DE3) cells were grown at 37 °C to OD600 of 0.6 to 0.8, induced with 1 mM IPTG, and left to shake at 18 °C overnight. Cells were harvested by centrifugation and resuspended in buffer A [50 mM Tris (pH 8.0) and 100 mM NaCl] and lysed by sonication. The clarified lysate was loaded onto a nickel-charged chelating column, washed with buffer A, and eluted with buffer B [50 mM Tris (pH 8.0), 100 mM NaCl and 1 M imidazole]. The eluent was loaded onto an SP column, washed with buffer C [50 mM Tris (pH 8.0), 1 mM EDTA, 10% glycerol, 100 mM NaCl], and eluted in buffer D [50 mM Tris (pH 8.0), 1 mM EDTA, 10% glycerol, 1 M NaCl]. Protein concentration was estimated using ε_280_ of 37,000 M^−1^ cm^−1^ as the extinction coefficient for OGG1.

### DNA substrates

Oligonucleotides were purchased from the Keck Oligo Synthesis Resource at Yale University and gel purified. The following sequences were used in this study: 5’ CGTTCAACGTGCACTXACAGCACGTCCCAT and the complementary 5’ ATGGGACGTGCTGTYAGTGCACGTTGAACG (X is either 8-oxoG or THF and Y is either a templating cytosine or adenosine). The lesion-containing DNA strand was phosphorylated at the 5’ terminus using [γ-^32^P]ATP and T4 polynucleotide kinase. Excess ATP was removed by a Microspin column. The radiolabeled strand and the complementary strand were annealed in 50 mM Tris-HCl (pH 7.6), 50 mM KCl, 1 mM EDTA, and 10% glycerol to generate a double-stranded DNA substrate with the lesion. Annealing was confirmed using a native gel.

### Electrophoretic mobility shift assay

DNA containing either 8-oxoG or THF (0.1 nM) was incubated with OGG1 at a concentration range of 0.5 to 4000 nM for 15 min at 4 °C in 25 mM Tris-HCl (pH 7.6), 175 nM NaCl, 1 mM EDTA, 1 mM DTT, 300 μg/ml bovine serum albumin, and 12% glycerol. Free DNA was separated from bound DNA by running the samples on a 10% native gel in Tris/Borate/EDTA buffer at 150 V for 2 h at 4 °C. Fraction-bound DNA (Y) was plotted as a function of OGG1 concentration (X). Dissociation constants (*K*_d_) were extracted using Equation 1, where *B*_max_ is the maximum bound fraction.

(1) $$Y=B_{max}*\frac{X}{K_{d}+X}+Background$$

### Glycosylase activity assay

Reactions were initiated by rapidly mixing 500 nM OGG1 with equal volumes of 50 nM DNA (final concentrations) at 37 °C in 50 mM Tris-HCl (pH 8.0), 20 mM NaCl, 2 mM DTT, and 10% glycerol. The reaction was quenched using a KinTek rapid quench-flow apparatus for short time points and by hand for time points longer that 120 s. The abasic site formed as a result of the glycosylase activity was quenched with 700 mM NaOH and then heated at 85 °C for 5 min. Reaction product was separated by gel electrophoresis using a 20% polyacrylamide gel containing 6 M urea. Gels were exposed to a phosphor screen, and the signal was detected by phosphorescence emission. The intensities of the product and substrate bands were quantified using ImageQuant (GE Healthcare), and the fraction product formed was plotted as a function of time (*t*). Points were fitted to a single-exponential equation (Equation 2) using GraphPad Prism to obtain an observed rate (*k*_obs_) for each of the mutants.

(2) $$\text{Fraction}\;\text{product}=A\left(1-e^{-k\text{obs}}t\right)$$

A is the amplitude of the reaction. Reactions were performed in triplicates and reported as mean ± standard error.

### Lyase activity assay

Reactions were performed by hand similarly to the glycosylase assay and quenched with 90% formamide containing bromophenol blue. Product analysis was performed as described above.

### Circular dichroism

The secondary structure of 6 μM OGG1 in 10 mM dibasic sodium phosphate was measured using the Applied Photophysics circular dichroism spectrometer at 195 to 250 nm. Thermal stability of the secondary structure was measured at 208 nm from 25 to 55 °C, and the melting temperature (*T*_m_) was estimated by fitting the points to a sigmoidal equation.

## Data availability

All data are contained within the article. Raw gels are also available upon request.

## Conflict of interest

The authors declare that they have no conflicts of interest with the contents of this article.
